# HIF-2α regulates proliferation, invasion, and metastasis of hepatocellular carcinoma cells *via* VEGF/Notch1 signaling axis after insufficient radiofrequency ablation

**DOI:** 10.3389/fonc.2022.998295

**Published:** 2022-09-23

**Authors:** Yongguang Yang, Weifeng Chen, Weiheng Mai, Yi Gao

**Affiliations:** ^1^ Second Department of Hepatobiliary Surgery, Guangdong Provincial, Research Center for Artificial Organ and Tissue Engineering, Guangzhou Clinical Research and Transformation Center for Artificial Liver, Institute of Regenerative Medicine, Zhujiang Hospital, Southern Medical University, Guangzhou, China; ^2^ State Key Laboratory of Organ Failure Research, Southern Medical University, Guangzhou, China; ^3^ Department of Hepatobiliary Surgery, The Affiliated Hospital of Guangdong Medical University, Zhanjiang, China

**Keywords:** hepatocellular carcinoma, residual carcinoma, radiofrequency ablation, hypoxia-inducible factor-2α, metastasis

## Abstract

**Background and Aims:**

Although insufficient radiofrequency ablation (RFA) promotes the recurrence and metastasis of liver cancer, the underlying mechanism remains unclear. This study aimed to investigate the role and mechanism of HIF-2α in hepatocellular carcinoma cells (HCCs) after Insufficient RFA.

**Methods:**

We established a model of insufficient RFA in MHCC97H hepatoma cells and screened for stable sublines. We inhibited HIF-2α expression in the Insufficient RFA group using PT2385 and assessed the resulting changes in proliferation and biological function of HCCs. Cell viability and proliferation were detected by the MTT method, and scratch and Transwell chamber invasion tests detected migration and invasion abilities of HCCs. The mRNA and protein expression levels of VEGF, HIF-2α, and Notch1 were detected using qPCR, immunofluorescence, and western blotting.

**Results:**

Compared with normal HCCs without RFA treatment, insufficient RFA enhanced the proliferation and invasion abilities of hepatocellular carcinoma subline MHCC97H (*P* < 0.001), as well as their migration ability (*P* = 0.046). The HIF-2α-specific inhibitor PT2385 downregulated the migration (*P* = 0.009) and invasion (*P* < 0.001) of MHCC97H cells but did not affect cell proliferation (*P* > 0.05). Insufficient ablation increased the mRNA and protein expression of VEGF, HIF-2α, and Notch1 in HCCs, whereas inhibition of HIF-2α reversed these changes.

**Conclusions:**

Insufficient RFA increases the proliferation, migration, and invasion of HCCs *via* the HIF-2α/VEGF/Notch1 signaling axis; HIF-2α is a potential target for novel treatments of HCC after insufficient RFA.

## Introduction

Primary liver cancer is the sixth most common cancer and was the third leading cause of cancer death globally in 2020, with approximately 906,000 new cases and 830,000 deaths, of which 410,038 new cases and 391,152 deaths occurred in China, accounting for 45 and 47% of the totals, respectively, ranking first in the world (1). Liver cancer is highly malignant and develops rapidly. The early symptoms of liver cancer are not obvious; most patients with liver cancer are in the middle and late stages when diagnosed (2). Currently, the methods for treating liver cancer include hepatectomy, liver transplantation, and local ablation (3). However, the lack of liver donors seriously restricts the clinical application of liver transplantation. Hepatectomy is still the most effective treatment for liver cancer worldwide. However, liver cancer in most patients in China is accompanied by cirrhosis and portal hypertension, elevating the risk of liver failure after hepatectomy. In recent years, radiofrequency ablation (RFA) has become an indispensable tool in treating liver cancer owing to its advantages of being minimally invasive, economical, simple, and repeatable, as well as its low damage to surrounding liver tissue and high safety (4).

RFA works by transmitting electrical energy to the top of the electrode needle through a radiofrequency field formed by a closed circuit between a radiofrequency generator and the patient. When energized, the tumor tissue between the electrodes blocks the conduction of electricity, which generates heat and high temperatures. Finally, the tumor achieves coagulation, necrosis and inactivation (5). However, tumor size, shape, and location complicate the application of RFA and may result in insufficient tumor ablation. Liver cancer patients with insufficient RFA have a high risk of recurrence, metastasis, and disease progression (6–8), though the underlying mechanisms remain unknown (9). Although RFA triggers coagulation necrosis of some hepatocellular carcinoma cells (HCCs), residual cells increase the expression of vascular endothelial growth factor (VEGF) to promote endothelial cell proliferation in the hypoxic tumor microenvironment. Tumor-associated angiogenesis increases the recurrence and metastasis of liver cancer after RFA treatment. In previous studies by our group and other experts, the activation of neurogenic locus notch homolog protein 1 (Notch1) signaling was found to play a vital role in the proliferation of residual carcinoma cells (10, 11). Therefore, VEGF-mediated tumor-associated angiogenesis and activated Notch1 signal-driven tumor survival are among the most important molecular mechanisms contributing to HCC recurrence and progression.

It is well accepted that hypoxia-inducible factor (HIF) is elevated in residual carcinoma cells after RFA, including HIF-1α and HIF-2α (12). Overexpression of HIF-1α or HIF-2α has been detected in patients with HCC and has been closely associated with poor clinical outcomes (13). When subjected to persistent hypoxic stimulation, residual carcinoma expresses more HIF-2α than HIF-1α (14–17). HIF-2α is a key activator of the hypoxia response and is higher than HIF-1α in the transcriptional regulation of genes related to angiogenesis, angiogenesis, invasion, and metastasis (18). Moreover, HIF-2α promotes VEGF expression to a greater degree than HIF-1α (19, 20). Few studies have reported that HIF-2α is a crucial upstream regulator of VEGF and Notch1 signaling. However, the functional role of HIF-2α in HCC recurrence after insufficient RFA remains unclear. This study aimed to explore the role of HIF-2α in an *in vitro* insufficient RFA cell model.

## Materials and methods

### Reagents and chemicals

Our study incorporated the following reagents/materials at different stages: DMEM medium, fetal bovine serum (Gibco, USA), tetrazolium blue (MTT) powder (Dongguan Science and Technology Biology Company), Transwell chambers, Matrigel matrix glue (Corning, USA), mouse anti-VEGF monoclonal antibody (Proteintech), rabbit anti-human HIF-2α, rabbit anti-human Notch1, rabbit anti-human β-actin (monoclonal antibodies; CST), and PT2385 (MCE). Shanghai Shenggong Bioengineering Company synthesized the PCR primers. The reverse transcription kit and fluorescence quantitative PCR kit were obtained from Takara Bio.

### Clinical samples

Cancerous and paracancerous tissues were obtained from six patients (with complete clinical records) with HCC treated by RFA between June 2018 and June 2021 in the Department of Hepatobiliary Surgery, Affiliated Hospital of Guangdong Medical University (Zhanjiang, China). All patients were diagnosed with HCC by the pathology department after surgery. Insufficient RFA was diagnosed after one month by arterial contrast enhancement and port venous washout within the RFA site suggestive of residual tumor tissue on enhanced CT or MR imaging, confirmed by pathology. The ethics committee of the Affiliated Hospital of Guangdong Medical University approved this study (LCYJ2021B002), and written informed consent was obtained from all patients.

### Immunohistochemistry

Tissue samples were harvested and fixed in 10% formaldehyde (pH 7.4), dehydrated, and embedded in paraffin. We deparaffinized and rehydrated 4-μm sections of the paraffin-embedded tissue, performed epitope retrieval and blockade of endogenous peroxidase, incubated the sections with primary and HRP-conjugated secondary antibodies, followed by DAB immunostaining and hematoxylin counterstaining. Images were obtained using a light microscope equipped with a DP74 digital camera (Olympus, Japan).

### Cell culture and establishment of insufficient RFA cell model

The HCC cell line MHCC97H (NC) was purchased from the cell bank of Chinese Academy of Science (Shanghai, China), and cells were cultured in DMEM supplemented with 10% FBS containing 100 U/mL penicillin and streptomycin at 37°C and 5% CO2. The medium was changed once daily. The cells were digested with 0.25% trypsin and separated into single-cell suspensions for passaging.

To mimic RFA treatment *in vitro*, MHCC97H cells were seeded in a 96-well plate and cultured at 47°C for 10 min. The surviving cells were named MHCC97H-H (NC-H) and their evaluation confirmed insufficient RFA (21). Different doses (10 nM to 100 μM) of the selective HIF-2α inhibitor PT2385 were tested to determine the optimal concentration for further analysis.

### MTT cell proliferation assay

MTT cell proliferation assay kit (Dongguan Science and Technology Biology Company) was performed according to the manufacturer’s instructions. The optical density was measured using a multimode reader at 492 nM.

### Wound healing assay

The 10^5^ cells of suspension were added to a 6-well plate with inserts in place and then cultured until a monolayer was formed. A wound was created by scraping the monolayer with a 1 mL pipette tip. The cells were washed once and the medium was replaced. The cells were monitored for migration into the wound field after a 24-h culture. The results were observed using an inverted microscope with phase contrast.

### Invasion/migration assay

Matrigel was added on top of the membrane of a 24-well Transwell plate and solidified in a 37°C incubator for 15–30 min to form a thin gel layer. Cells in the logarithmic growth phase were digested with trypsin and resuspended in a serum-free medium. A cell suspension (200μL) was added to the upper chamber of the Transwell insert and 600 μL medium containing 20% FBS was added to the bottom chamber. After culturing for 24-h, the Transwell insert was fixed with methanol for 15 min and then stained with crystal violet. The upper layer of the unmigrated cells was gently wiped with a cotton swab (22). Ten random fields were photographed under a microscope at 200× magnification. We considered the relative number of invasive cells to correspond with the migratory ability of the tumor cells.

### Real-time quantitative PCR

Total RNA from cultured cells was extracted using TRIzol reagent. Complementary DNA was synthesized using the M-MuLV First Strand cDNA Synthesis Kit (Sangon Biotech, Shanghai, China), and real-time PCR was performed as previously described (23), using the following primers: human HIF-2α (Forward 5′-GTCATCTACAACCCTCGCAACCTG-3′, reverse 5′-ACCACGTCATTCTTCTCAATCTCACTC-3′), human Notch1 (Forward 5′-ACCACTGCGAGACCAACATCAAC-3′, Reverse 5′-CAGAAGCAGAGGTAGGCGTTGTC-3′), human VEGF (Forward 5′-CGAAACCATGAACTTTCTGC-3′, Reverse 5′- CCTGAGTGGGCACACACTCC-3′), and human glycolytic glyceraldehyde-3-phosphate dehydrogenase (GAPDH, Forward 5′-ACATCGCTCAGACACCATG-3′, reverse 5′-TGTAGTTGAGGTCAATGAAGGG-3′).

### Western blotting

Western blotting was used to detect the expression of target proteins. Protein samples were extracted by RIPA lysis buffer and subjected to 12% sodium dodecyl sulfate-polyacrylamide gel electrophoresis. All proteins were transferred from sodium dodecyl sulfate-polyacrylamide gel electrophoresis to sodium dodecyl sulfate-polyacrylamide gel electrophoresis, followed by incubation with the primary and HRP-conjugated secondary antibodies. GAPDH was used as the loading control. The integrated optical density and the area of the protein bands were quantified and analyzed using ImageJ software (National Institutes of Health, Bethesda, MD, USA).

### Statistical analysis

Data are shown as mean ± standard error of the mean (SEM) from at least three independent experiments. The student’s *t*-test was used for between-group comparisons. One-way analysis of variance was used for comparisons among multiple groups, followed by Tukey’s *post hoc* test. Statistical significance was set at p < 0.05. Data analysis was performed, and graphics were created using GraphPad Prism 5 (GraphPad Software, San Diego, CA, USA).

## Results

### HIF-2α expression in HCC and their paracancerous tissues after insufficient RFA

**Figure 1:**
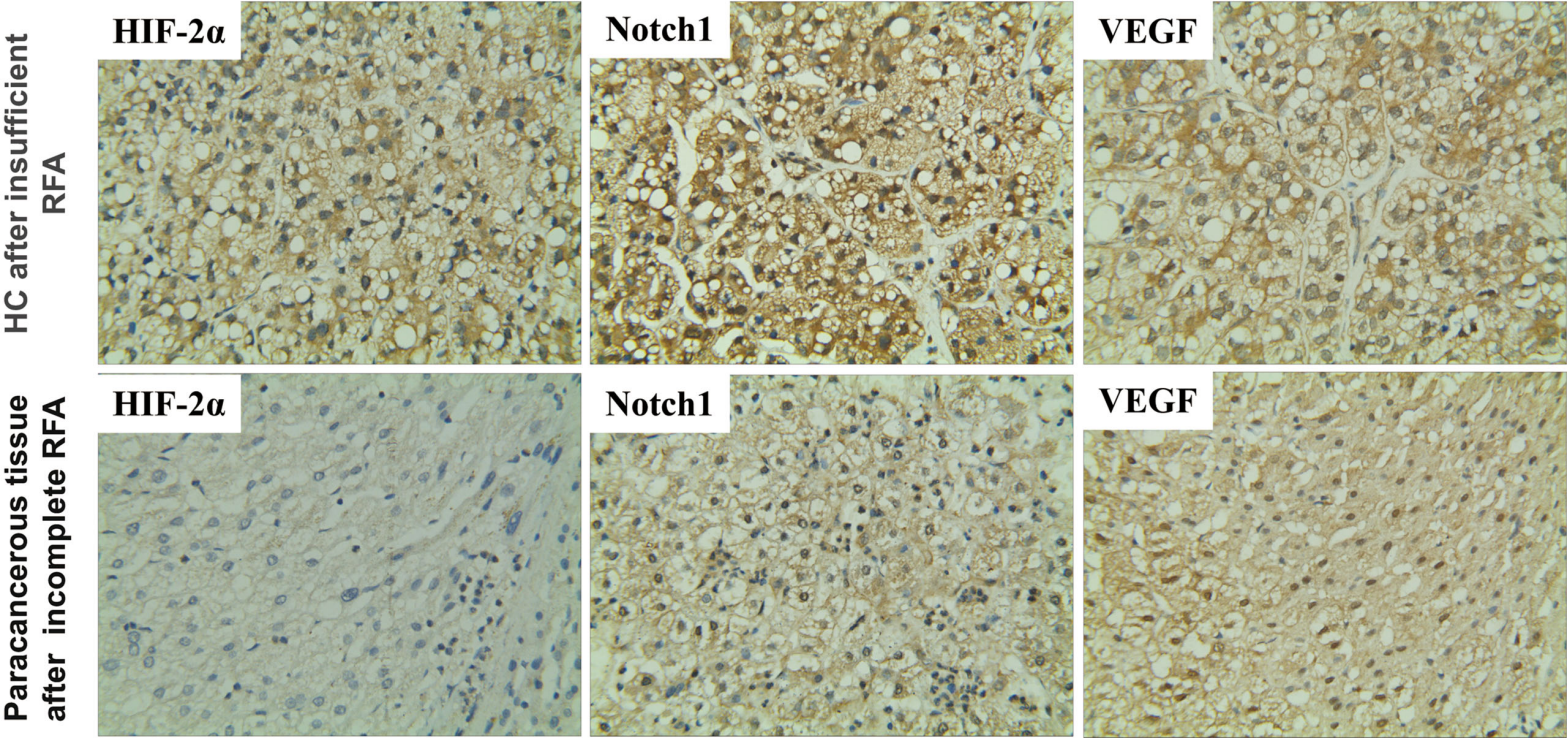
Expression of HIF-2α, Notch1, and VEGF in HC and their paracancerous tissues of patients after insufficient RFA as revealed by immunohistochemistry. Brown color indicates positive staining. Magnification 400×.

First, we tested whether HIF-2α is involved in the recurrence of patients with HCC after RFA. Immunohistochemistry revealed that the expression of HIF-2α was higher in cancerous than paracancerous tissues and was mainly concentrated to the cytoplasm (**Figure 1**). The levels of Notch1 and VEGF were elevated similarly **(** **Figure 1**). These data indicate that HIF-2α, Notch1, and VEGF may be involved in HC recurrence after insufficient RFA.

### Insufficient RFA promoted the invasion and proliferation of HCCs

**Figure 2:**
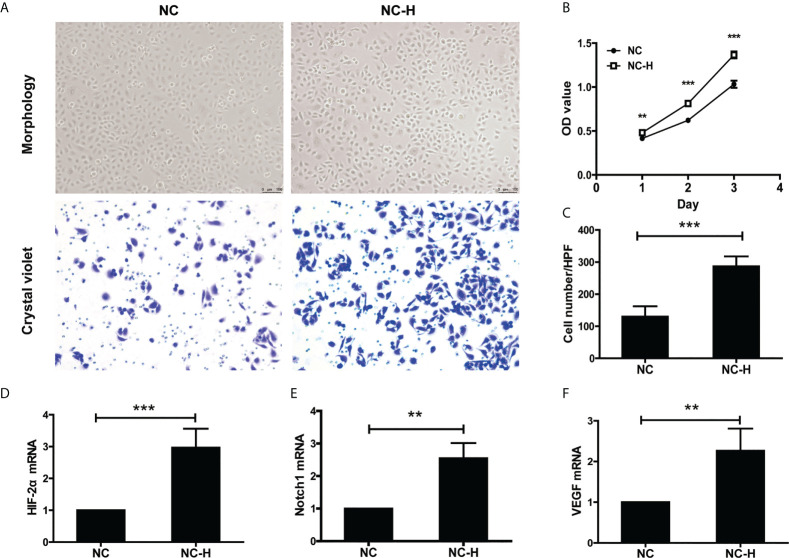
HIF-2α, Notch1, and VEGF were involved in the increased invasion and proliferation of HCC induced by insufficient RFA. **(A)** Representative photos of HCC after insufficient RFA were detected by a phase contrast microscope and crystal violet staining. Magnification 400×. **(B)** Detection of cell proliferation by MTT. **(C)** Quantitation analysis of Transwell assay. **(D–F)** Detection of the mRNA expression of HIF-2α, Notch1, and VEGF. **P < 0.01, ***P < 0.001.

Then, we established an insufficient RFA Cell Model according to the previous study (17). We performed MTT and Transwell assays to investigate the effect of insufficient RFA on HCC proliferation and invasion. After being subjected to a sub-lethal heat shock, NC-H cells displayed a fusiform shape and proliferated rapidly (**Figures 2A, B** **)**. The Transwell assay showed insufficient RFA treatment enhanced NC-H invasion ability (**Figures 2A, C** **)**. In addition, the mRNA expression of HIF-2α, VEGF, and Notch1 was markedly increased after insufficient RFA treatment (**Figures 2D** **–F**).

### Inhibition of HIF-2α by PT2385 suppressed the invasion and migration of HCCs

**Figure 3:**
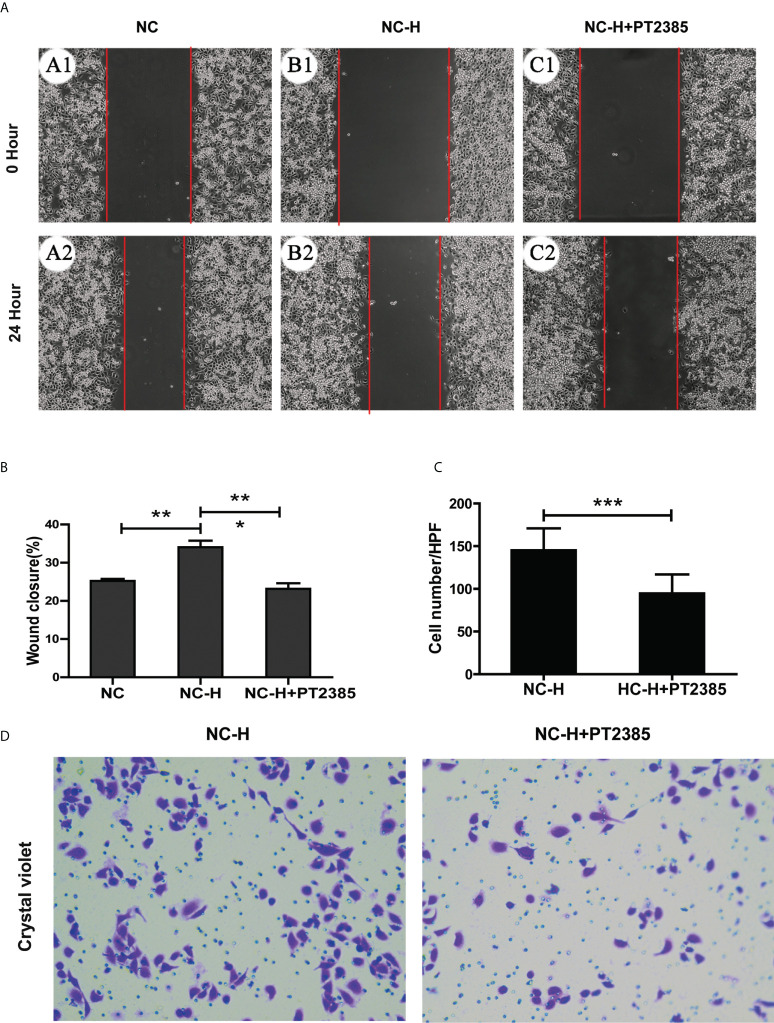
Inhibition of HIF-2α by PT2385 suppressed the invasion and migration of HCCs after insufficient RFA. **(A)** Representative photos of HCC after insufficient RFA were detected by wound healing assay. Magnification 200×. **(B)** Quantitation analysis of wound healing assay. **(C, D)** Representative photos and quantitation analysis of Transwell assay. Magnification 400×. *P < 0.05; **P < 0.01. ***P < 0.001.

To assess the role of HIF-2α in elevating the invasion and migration ability of HCCs after insufficient RFA, we performed Transwell and wound healing assays. We found that the increased migration ability of NC-H was suppressed by PT2385, a selective antagonist of HIF-2α over HIF-1α (**Figures 3A, B** **)**. Similarly, PT2385 also inhibited the enhanced invasive ability of NC-H, as indicated by the Transwell assay (**Figures 3C, D** **)**.

### Inhibition of HIF-2α by PT2385 suppressed the VEGF and Notch1 signaling pathway

**Figure 4:**
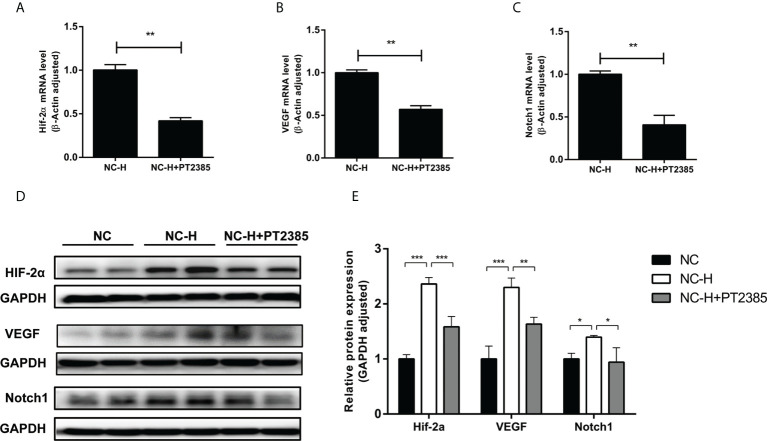
Inhibition of HIF-2α by PT2385 suppressed the VEGF and Notch1 signaling pathway in HCC after insufficient RFA. **(A–C)** Detection of mRNA expression by RT-qPCR. **(D, E)** Detection of protein levels by western blotting. *P < 0.05, **P < 0.01, ***P < 0.001.

Finally, we tried to explore a potential pathway by which HIF-2α inhibition suppressed the invasion and migration of HCCs. As expected, PT2385 inhibited the expression of HIF-2α at both the mRNA and protein levels **(** **Figures 4A, D, E** **)**. Interestingly, inhibition of HIF-2α by PT2385 also notably suppressed the expression of VEGF and Notch1, as detected by RT-qPCR and western blotting **(** **Figures 4B** **–E** **)**.

## Discussion

HCC is one of the most common malignant tumors, and its fatality rate ranks second among all malignant tumors worldwide (24), claiming 380,000 lives each year and accounting for half the cancer-related deaths in China (25–27). Hepatectomy, liver transplantation, and local ablative therapy are available treatments, but there is still a high incidence of postoperative recurrence (28). RFA has various advantages and is an important clinical treatment for liver cancer. It works by generating heat and high temperature to induce tumor tissue coagulation, necrosis, and inactivation. In general, 46°C for 60 min can lead to irreversible cell damage, and the higher the temperature, the shorter the ablation time. However, when the local temperature exceeds 105°C, tumor tissue vaporization will occur, increasing the total resistance of radiofrequency energy, resulting in RFA insufficiency. Therefore, the appropriate temperature for RFA is 50 to 100°C. Ablation is mainly suitable for single tumors with a diameter of less than 5cm or two to three tumors which a maximum diameter of less than 3 cm. Uni- or multipolar needles are clinically used depending on liver tumor size and location. The former is used for tumors with a diameter of less than 3 cm and the latter for tumors with a diameter of 3 to 5 cm. The ablation scope is generally extended to more than 1 cm of the tumor diameter to ensure tumor tissue destruction. For tumors with a diameter of up to 3 cm, one to two rounds of superimposed treatment for 5 mins is suggested, while 10 min of six treatments is recommended for tumor diameters between 3 and 4 cm. For tumors with a diameter above 4 cm, multiple overlapping treatments should be performed for 10 to 15 min (29).

However, due to the heterogeneity in tumor characteristics, ablation rates vary between 10.3–38.7% (30). Multi-point combined thermal field ablation can be used when the tumor diameter is 3 to 5 cm. Gasification occurs during the tissue carbonization and necrosis process, interfering with observation. When blind areas are left and there is no overlap between each ablation area, insufficient tumor ablation occurs. When the tumor is adjacent to large blood vessels, blood flux will draw part of the heat induced by RFA. Due to the reduced treatment temperature, the cancer cells near large blood vessels are preserved, resulting in insufficient RFA (31, 32). When the tumor is located on the surface of the liver or adjacent to Gleason’s pedicle, ablation may cause damage to the gastrointestinal tract, diaphragm, kidney, or biliary tract. Therefore, the scope of ablation is limited to avoid or reduce damage to special organs and vessels, resulting in residual tumor cells (33, 34). In addition, patients subjected to insufficient RFA often present local tumor recurrence and metastasis in the short term and even experience rapid deterioration in health (35–38). The potential harm that this technology can cause is the biggest obstacle to its successful application (9). Cell invasion and metastasis after insufficient RFA have recently gained attention (39–41), but the mechanism remains unclear.

Notch signaling is a conserved and important pathway involved in proliferation, differentiation, and self-renewal in most cell types (42, 43). It includes Notch ligands (DLL1, DLL3 DLL4, Jagged1, and Jagged2), Notch receptors (Notch1, -2, -3, and -4), and downstream target genes (Hes and Hey) (44). Our group and others have found that activation of the Notch1 pathway contributes to cancer cell proliferation, invasion, and migration (11, 45–47) and drug resistance in tumors (48). Notch1 mRNA and protein expression were elevated in HCC, and targeting Notch1/Hes1 using dihydromyricetin suppressed HCC proliferation and induced HCC apoptosis (11). Moreover, the level of Notch1 was increased in the liver tumor tissue of patients after insufficient RFA and in *in vitro* cell models, indicating that Notch1 signaling may be associated with the recurrence of HCC after insufficient RFA.

VEGF is also involved in HC recurrence after insufficient RFA. It is well known that VEGF stimulates endothelial cell growth and migration and increases vascular permeability and endothelial cell activity. VEGF expression is increased in various cancers, including HCC, and is associated with the invasion, recurrence, metastasis, and prognosis of liver cancer. Although they show some therapeutic effects, tyrosine kinase inhibitors targeting the VEGF receptor cause cardiotoxicity, hypertension, hand-foot syndrome, and other side effects. Recently, VEGF has been reported to activate the Notch1 pathway by upregulating DLL4 expression. The blocking of the Notch signaling pathway through DLL4-and VEGF acts synergistically to reduce the density and function of tumor vessels and inhibit tumor growth (49). Similarly, we found that the expression levels of VEGF and Notch1 were elevated after insufficient RFA *in vivo* and *in vitro*. Thus, there is an urgent need to identify upstream molecules that regulate VEGF and Notch1, which may provide a strategy for personalized therapies.

Hypoxia, the insufficient supply of oxygen to tissues, is an inherent characteristic of the tumor microenvironment and exists in almost all solid tumor sites (50). Tumor hypoxia leads to the activation of the HIF signaling pathway, and HIF is involved in mediating many important processes, such as tumor growth, metastasis, metabolism, and angiogenesis (51). HIF is a heterodimer complex consisting of a HIF-α subunit degraded by an oxygen-dependent proteasome and a constitutively expressed HIF-β subunit (52). Low levels of HIF expression are observed in normal liver tissues. However, HIF accumulates in large quantities under hypoxic conditions and becomes more stable. HIF is involved in mediating growth, metastasis, metabolism, angiogenesis, drug resistance, and other essential processes in liver cancer (14, 53). HIF-1α is mainly associated with acute hypoxia in tumors, whereas HIF-2α plays a major role in long-term chronic hypoxia (15, 16, 20). In the current study, the increased expression of HIF-2α was accompanied by elevated VEGF and Notch1 expression after insufficient RFA *in vivo* and *in vitro*, indicating that it acts as a potential upstream mediator. Similarly, HIF-2α has been shown to promote angiogenesis *via* the VEGF/Notch pathway to attenuate intracerebral hemorrhage injury (54). PT-2385 is inactive against HIF-1α and is a selective HIF-2α inhibitor with a Ki of less than 50 nM (55). The HIF-2α antagonist PT2385 exhibited a significant therapeutic effect in the phase I clinical trials of other tumor types such as human renal clear cell carcinoma and did not cause side effects such as cardiotoxicity and hypertension (56). In addition, PT2385 has been authorized for production by Peloton, which is convenient for experimental research. Thus, our results on HCC after insufficient RFA are amenable to clinical translation. Targeting HIF-2α with PT2385 attenuated renal cell carcinoma progression more effectively than sunitinib, accompanied by better tolerance and fewer side effects (56, 57). In our study, inhibition of HIF-2α suppressed the enhanced invasion and migration abilities of HCC after insufficient RFA. Moreover, the increased expression of VEGF and Notch1 was downregulated following PT2385 treatment. Similarly, PT2385 has been reported to suppress VEGF mRNA expression *via* HIF-2α inhibition in renal cell carcinoma and hypoxic HCCs (58). A previous study reported that HIF-2α repressed Notch signaling, but HIF-1α promoted it (59). In contrast, other scholars found that HIF-2α overexpression increased the activation of Notch pathways (60). In our study, the elevated expression of Notch1 may have directly resulted from increased VEGF expression, with HIF-2α having an indirect effect. However, the detailed mechanism of action of the PT2385 regulation of Notch1 expression needs to be explored in further studies.

In summary, our study revealed that insufficient RFA induced the activation of the HIF-2α/VEGF/Notch1 signaling axis in HCC, leading to enhanced proliferation, migration, and invasion of HCCs. Furthermore, HIF-2α is a potential upstream regulatory molecule of the VEGF and Notch1 pathways, though further research is required. To our knowledge, this is the first study determining the potential of PT2385 in treating HCC after insufficient RFA.

## Data availability statement

The raw data supporting the conclusions of this article will be made available by the authors, without undue reservation.

## Ethics statement

The Ethics Committee approved this study of the Affiliated Hospital of Guangdong Medical University. The patients/participants provided their written informed consent to participate in this study.

## Author contributions

All authors read and approved the final manuscript. YY established insufficient radiofrequency ablation model of hepatocellular carcinoma cells. YY, WC, and MW completed the changes in cell proliferation, migration, and invasion and the tests of Q-PCR, immunofluorescence, and Western blot. YY and WF wrote the original draft. YG designed the study, supervised research, and edited the manuscript. All authors contributed to the article and approved the submitted version.

## Funding

This study was supported by the National Key R&D Program of China (2018YFA0108200; 2018YFC1106400); The National Natural Science Foundation of China (31972926); the Science Foundation of Guangdong Province (2018A030307076); Zhanjiang City Financial Fund Technology Special Competitive Allocation Project (2018A01037); National Natural Science Foundation of China “Breakthrough” Support Project (20301DFY20190157); Scientific research project of Guangdong Provincial Bureau of Traditional Chinese Medicine (20201189).

## Acknowledgments

We are appreciated for support from the imaging department and pathology during the patient’s diagnosis, treatment, and our draft process. We are equally appreciated for the patient’s cooperation in our medical activity and the generous authorization of our report. Finally, we would like to thank Editage (www.editage.cn) for English language editing.

## Conflict of interest

The authors declare that the research was conducted in the absence of any commercial or financial relationships that could be construed as a potential conflict of interest.

## Publisher’s note

All claims expressed in this article are solely those of the authors and do not necessarily represent those of their affiliated organizations, or those of the publisher, the editors and the reviewers. Any product that may be evaluated in this article, or claim that may be made by its manufacturer, is not guaranteed or endorsed by the publisher.

## References

[1] Sung H Ferlay J Siegel RL Laversanne M Soerjomataram I Jemal A . Global cancer statistics 2020: GLOBOCAN estimates of incidence and mortality worldwide for 36 cancers in 185 countries. CA Cancer J Clin (2021) 71(3):209–49. doi: 10.3322/caac.21660 33538338

[2] Yang WS Zeng XF Liu ZN Zhao QH Tan YT Gao J . Diet and liver cancer risk: a narrative review of epidemiological evidence. Br J Nutr (2020) 124(3):330–40. doi: 10.1017/S0007114520001208 32234090

[3] Lee SK Lee SW Jang JW Bae SH Choi JY Yoon SK . Immunological markers, prognostic factors and challenges following curative treatments for hepatocellular carcinoma. Int J Mol Sci (2021) 22(19):10271. doi: 10.3390/ijms221910271 34638613 PMC8508906

[4] Rossi S Fornari F Pathies C Buscarini L . Thermal lesions induced by 480 KHz localized current field in guinea pig and pig liver. Tumori (1990) 76(1):54–7. doi: 10.1089/sct.1990.6.51 2181746

[5] Zervas NT Kuwayama A . Pathological characteristics of experimental thermal lesions. comparison of induction heating and radiofrequency electrocoagulation. J Neurosurg (1972) 37(4):418–22. doi: 10.3171/jns.1972.37.4.0418 4560953

[6] Su T Liao J Dai Z Xu L Chen S Wang Y . Stress-induced phosphoprotein 1 mediates hepatocellular carcinoma metastasis after insufficient radiofrequency ablation. Oncogene (2018) 37(26):3514–27. doi: 10.1038/s41388-018-0169-4 29559743

[7] Cheng J Li M Lv Y . Sublethal heat treatment promotes epithelial-mesenchymal transition and enhances the malignant potential of hepatocellular carcinoma. Hepatology (2014) 59(4):1650. doi: 10.1002/hep.26630 23908073

[8] Sun C Bai M Ke W Wang X Zhao X Lu Z . The HSP90 inhibitor, XL888, enhanced cell apoptosis *via* downregulating STAT3 after insufficient radiofrequency ablation in hepatocellular carcinoma. Life Sci (2021) 282:119762. doi: 10.1016/j.lfs.2021.119762 34186047

[9] Nijkamp MW van der Bilt JD de Bruijn MT Molenaar IQ Voest EE van Diest PJ . Accelerated perinecrotic outgrowth of colorectal liver metastases following radiofrequency ablation is a hypoxia-driven phenomenon. Ann Surg (2009) 249(5):814–23. doi: 10.1097/SLA.0b013e3181a38ef5 19387320

[10] Kunnimalaiyaan S Gamblin TC Kunnimalaiyaan M . Glycogen synthase kinase-3 inhibitor AR-A014418 suppresses pancreatic cancer cell growth via inhibition of GSK-3-mediated Notch1 expression. HPB (Oxford) (2015) 17(9):770–6. doi: 10.1111/hpb.12442 PMC4557650 26147011

[11] Lu CJ He YF Yuan WZ Xiang LJ Zhang J Liang YR . Dihydromyricetin-mediated inhibition of the Notch1 pathway induces apoptosis in QGY7701 and HepG2 hepatoma cells. World J Gastroenterol (2017) 23(34):6242–51. doi: 10.3748/wjg.v23.i34.6242 PMC5603490 28974890

[12] Yang SL Liu LP Niu L Sun YF Yang XR Fan J . Downregulation and pro-apoptotic effect of hypoxia-inducible factor 2 alpha in hepatocellular carcinoma. Oncotarget (2016) 7(23):34571–81. doi: 10.18632/oncotarget.8952 PMC5085177 27119229

[13] Wilson GK Tennant DA McKeating JA . Hypoxia inducible factors in liver disease and hepatocellular carcinoma: current understanding and future directions. J Hepatol (2014) 61(6):1397–406. doi: 10.1016/j.jhep.2014.08.025 25157983

[14] Koh MY Lemos R Jr Liu X Powis G . The hypoxia-associated factor switches cells from HIF-1α- to HIF-2α-dependent signaling promoting stem cell characteristics, aggressive tumor growth and invasion. Cancer Res (2011) 71(11):4015–27. doi: 10.1158/0008-5472.CAN-10-4142 PMC3268651 21512133

[15] Menrad H Werno C Schmid T Copanaki E Deller T Dehne N . Roles of hypoxia-inducible factor-1alpha (HIF-1alpha) versus HIF-2alpha in the survival of hepatocellular tumor spheroids. Hepatology (2010) 51(6):2183–92. doi: 10.1002/hep.23597 20513003

[16] Zhao J Du F Shen G Zheng F Xu B . The role of hypoxia-inducible factor-2 in digestive system cancers. Cell Death Dis (2015) 6(1):e1600. doi: 10.1038/cddis.2014.565 25590810 PMC4669763

[17] Chen M Shu G Lv X Xu X Lu C Qiao E . HIF-2α-targeted interventional chemoembolization multifunctional microspheres for effective elimination of hepatocellular carcinoma. Biomaterials (2022) 284:121512. doi: 10.1016/j.biomaterials.2022.121512 35405577

[18] Wu L Zhou J Zhou W Huang XF Chen Q Wang W . Sorafenib blocks the activation of the HIF-2α/VEGFA/EphA2 pathway, and inhibits the rapid growth of residual liver cancer following high-intensity focused ultrasound therapy in vivo. Pathol Res Pract (2021) 220:153270. doi: 10.1016/j.prp.2020.153270 33640712

[19] Sun HX Xu Y Yang XR Wang WM Bai H Shi RY . Hypoxia inducible factor 2 alpha inhibits hepatocellular carcinoma growth through the transcription factor dimerization partner 3/ E2F transcription factor 1-dependent apoptotic pathway. Hepatology (2013) 57(3):1088–97. doi: 10.1002/hep.26188 PMC3594482 23212661

[20] Yao J Li J Geng P Li Y Chen H Zhu Y . Knockdown of a HIF-2α promoter upstream long noncoding RNA impairs colorectal cancer stem cell properties in vitro through HIF-2α downregulation. Onco Targets Ther (2015) 8:3467–74. doi: 10.2147/OTT.S81393 PMC4664519 26648739

[21] Obara K Matsumoto N Okamoto M Kobayashi M Ikeda H Takahashi H . Insufficient radiofrequency ablation therapy may induce further malignant transformation of hepatocellular carcinoma. Hepatol Int (2008) 2(1):116–23. doi: 10.1007/s12072-007-9040-3 PMC2716878 19669287

[22] Chen H Wu F Xu H Wei G Ding M Xu F . Centromere protein f promotes progression of hepatocellular carcinoma through ERK and cell cycle-associated pathways. Cancer Gene Ther (2022) 29(7):1033–42. doi: 10.1038/s41417-021-00404-7 34857915

[23] Lu C He Y Duan J Yang Y Zhong C Zhang J . Expression of Wnt3a in hepatocellular carcinoma and its effects on cell cycle and metastasis. Int J Oncol (2017) 51(4):1135–45. doi: 10.3892/ijo.2017.4112 PMC5592886 28902357

[24] Wallace MC Preen D Jeffrey GP Adams LA . The evolving epidemiology of hepatocellular carcinoma: a global perspective. Expert Rev Gastroenterol Hepatol (2015) 9(6):765–79. doi: 10.1586/17474124.2015.1028363 25827821

[25] Wang S Du X Han X Yang F Zhao J Li H . Influence of socioeconomic events on cause-specific mortality in urban shanghai, China, from 1974 to 2015: a population-based longitudinal study. CMAJ (2018) 190(39):E1153–61. doi: 10.1503/cmaj.180272 PMC6167223 30274992

[26] Jiang D Zhang L Liu W Ding Y Yin J Ren R . Trends in cancer mortality in China from 2004 to 2018: A nationwide longitudinal study. Cancer Commun (Lond) (2021) 41(10):1024–36. doi: 10.1002/cac2.12195 PMC8504142 34251754

[27] Chen W Zheng R Baade PD Zhang S Zeng H Bray F . Cancer statistics in China, 2015. CA Cancer J Clin (2016) 66(2):115–32. doi: 10.3322/caac.21338 26808342

[28] Bruix J Sherman M . American Association for the Study of Liver Diseases. Management of hepatocellular carcinoma: An update. Hepatology(2011) 53(3):1020–2. doi: 10.1002/hep.24199 PMC3084991 21374666

[29] Gazelle GS Goldberg SN Solbiati L Livraghi T . Tumor ablation with radio-frequency energy. Radiology (2000) 217(3):633–46. doi: 10.1148/radiology.217.3.r00dc26633 11110923

[30] Benetti A Berenzi A Gambarotti M Garrafa E Gelati M Dessy E . Transforming growth factor-beta1 and CD105 promote the migration of hepatocellular carcinoma-derived endothelium. Cancer Res (2008) 68(20):8626–34. doi: 10.1158/0008-5472.CAN-08-1218 18922939

[31] Feng K Yan J Li X Dong J . A randomized controlled trial of radiofrequency ablation and surgical resection in the treatment of small hepatocellular carcinoma. J Hepatol (2012) 57(4):794–802. doi: 10.1016/j.jhep.2012.05.007 22634125

[32] Koda M Murawaki Y Hirooka Y Kitamoto M Ono M Sakaeda H . Complications of radiofrequency ablation for hepatocellular carcinoma in a multicenter study: An analysis of 16346 treated nodules in 13283 patients. Hepatol Res (2012) 42(11):1058–64. doi: 10.1111/j.1872-034X.2012.01025.x 22583706

[33] Maeda M Saeki I Sakaida I Aikata H Araki Y Ogawa C . Complications after radiofrequency ablation for hepatocellular carcinoma: A multicenter study involving 9,411 Japanese patients. Liver Cancer (2020) 9(1):50–62. doi: 10.1159/000502744 32071909 PMC7024979

[34] Ogawa T Kawamoto H Kobayashi Y Nakamura S Miyatake H Harada R . Prevention of biliary complication in radiofrequency ablation for hepatocellular carcinoma-cooling effect by endoscopic nasobiliary drainage tube. Eur J Radiol (2010) 73(2):385–90. doi: 10.1016/j.ejrad.2008.10.021 19056192

[35] Dong S Kong J Kong F Kong J Gao J Ke S . Insufficient radiofrequency ablation promotes epithelial-mesenchymal transition of hepatocellular carcinoma cells through akt and ERK signaling pathways. J Transl Med (2013) 11(1):273. doi: 10.1186/1479-5876-11-273 24168056 PMC3842745

[36] Ke S Ding XM Kong J Gao J Wang SH Cheng Y . Low temperature of radiofrequency ablation at the target sites can facilitate rapid progression of residual hepatic VX2 carcinoma. J Transl Med (2010) 8:73. doi: 10.1186/1479-5876-8-73 20667141 PMC2917410

[37] Zhang N Li H Qin C Ma D Zhao Y Zhu W . Insufficient radiofrequency ablation promotes the metastasis of residual hepatocellular carcinoma cells *via* upregulating flotillin proteins. J Cancer Res Clin Oncol (2019) 145(4):895–907. doi: 10.1007/s00432-019-02852-z 30820716 PMC6435628

[38] Kong J Yao C Dong S Wu S Xu Y Li K . ICAM-1 activates platelets and promotes endothelial permeability through VE-cadherin after insufficient radiofrequency ablation. Adv Sci (Weinh) (2021) 8(4):2002228. doi: 10.1002/advs.202002228 33643788 PMC7887603

[39] Su T Huang M Liao J Lin S Yu P Yang J . Insufficient radiofrequency ablation promotes hepatocellular carcinoma metastasis through N6-methyladenosine mRNA methylation-dependent mechanism. Hepatology (2021) 74(3):1339–56. doi: 10.1002/hep.31766 33638162

[40] Tong Y Yang H Xu X Ruan J Liang M Wu J . Effect of a hypoxic microenvironment after radiofrequency ablation on residual hepatocellular cell migration and invasion. Cancer Sci (2017) 108(4):753–62. doi: 10.1111/cas.13191 PMC5406608 28182306

[41] Zhang Q Kong J Dong S Xu W Sun W . Metformin exhibits the anti-proliferation and anti-invasion effects in hepatocellular carcinoma cells after insufficient radiofrequency ablation. Cancer Cell Int (2017) 17:48. doi: 10.1186/s12935-017-0418-6 28450808 PMC5404300

[42] Maillard I Fang T Pear WS . Regulation of lymphoid development, differentiation, and function by the notch pathway. Annu Rev Immunol (2005) 23:945–74. doi: 10.1146/annurev.immunol.23.021704.115747 15771590

[43] Ferjentsik Z Hayashi S Dale JK Bessho Y Herreman A De Strooper B . Notch is a critical component of the mouse somitogenesis oscillator and is essential for the formation of the somites. PloS Genet (2009) 5(9):e1000662. doi: 10.1371/journal.pgen.1000662 19779553 PMC2739441

[44] Chillakuri CR Sheppard D Lea SM Handford PA . Notch receptor-ligand binding and activation: insights from molecular studies. Semin Cell Dev Biol (2012) 23(4):421–8. doi: 10.1016/j.semcdb PMC3415683 22326375

[45] Wang Z Li Y Banerjee S Kong D Ahmad A Nogueira V . Down-regulation of notch-1 and jagged-1 inhibits prostate cancer cell growth, migration and invasion, and induces apoptosis *via* inactivation of akt, mTOR, and NF-kappaB signaling pathways. J Cell Biochem (2010) 109:726–36. doi: 10.1002/jcb.22451 20052673

[46] Cui L Dong Y Wang X Zhao X Kong C Liu Y . Downregulation of long noncoding RNA SNHG1 inhibits cell proliferation, metastasis, and invasion by suppressing the notch-1 signaling pathway in pancreatic cancer. J Cell Biochem (2019) 120(4):6106–12. doi: 10.1002/jcb.27897 30520072

[47] Zhang Q Lu C Fang T Wang Y Hu W Qiao J . Notch3 functions as a regulator of cell self-renewal by interacting with the β-catenin pathway in hepatocellular carcinoma. Oncotarget (2015) 6(6):3669–79. doi: 10.18632/oncotarget.2898 PMC4414145 25668819

[48] Trindade A Djokovic D Gigante J Mendonça L Duarte A . Endothelial Dll4 overexpression reduces vascular response and inhibits tumor growth and metastasization in vivo. BMC Cancer (2017) 17(1):189–201. doi: 10.1186/s12885-017-3171-2 28288569 PMC5348880

[49] Zhang Y Zhang Y Wang J Gu H . Amarogentin inhibits liver cancer cell angiogenesis after insufficient radiofrequency ablation *via* affecting stemness and the p53-dependent VEGFA/Dll4/Notch1 pathway. BioMed Res Int (2020) 2020:5391058. doi: 10.1155/2020/5391058 33145353 PMC7596460

[50] Shao C Yang F Miao S Liu W Wang C Shu Y . Role of hypoxia-induced exosomes in tumor biology. Mol Cancer (2018) 17(1):120. doi: 10.1186/s12943-018-0869-y 30098600 PMC6087002

[51] Davis L Recktenwald M Hutt E Fuller S Briggs M Goel A . Targeting HIF-2α in the tumor microenvironment: Redefining the role of HIF-2α for solid cancer therapy. Cancers (Basel) (2022) 14(5):1259. doi: 10.3390/cancers14051259 35267567 PMC8909461

[52] Semenza GL . Hypoxia-inducible factors in physiology and medicine. Cell (2012) 148(3):399–408. doi: 10.1016/j.cell.2012.01.021 22304911 PMC3437543

[53] Arnaiz E Miar A Bridges E Prasad N Hatch SB Ebner D . Differential effects of HIF2α antagonist and HIF2α silencing in renal cancer and sensitivity to repurposed drugs. BMC Cancer (2021) 21(1):896. doi: 10.1186/s12885-021-08616-8 34353313 PMC8344147

[54] Chen H Xiao H Gan H Zhang L Wang L Li S . Hypoxia-inducible factor 2α exerts neuroprotective effects by promoting angiogenesis *via* the VEGF/Notch pathway after intracerebral hemorrhage injury in rats. Neuroscience (2020) 448:206–18. doi: 10.1016/j.neuroscience.2020.07.010 32736070

[55] Courtney KD Ma Y Diaz de Leon A Christie A Xie Z Woolford L . HIF-2 complex dissociation, target inhibition, and acquired resistance with PT2385, a first-in-Class HIF-2 inhibitor, in patients with clear cell renal cell carcinoma. Clin Cancer Res (2020) 26(4):793–803. doi: 10.1158/1078-0432.CCR-19-1459 31727677 PMC7024660

[56] Courtney KD Infante JR Lam ET Figlin RA Rini BI Brugarolas J . Phase I dose-escalation trial of PT2385, a first-in-Class hypoxia-inducible factor-2α antagonist in patients with previously treated advanced clear cell renal cell carcinoma. J Clin Oncol (2018) 36(9):867–74. doi: 10.1200/JCO.2017.74.2627 PMC5946714 29257710

[57] Chen W Hill H Christie A Kim MS Holloman E Pavia-Jimenez A . Targeting renal cell carcinoma with a HIF-2 antagonist. Nature (2016) 539(7627):112–7. doi: 10.1038/nature19796 PMC5340502 27595394

[58] Wallace EM Rizzi JP Han G Wehn PM Cao Z Du X . A small-molecule antagonist of HIF2α is efficacious in preclinical models of renal cell carcinoma. Cancer Res (2016) 76(18):5491–500. doi: 10.1158/0008-5472.CAN-16-0473 27635045

[59] Hu YY Fu LA Li SZ Chen Y Li JC Han J . Hif-1α and hif-2α differentially regulate notch signaling through competitive interaction with the intracellular domain of notch receptors in glioma stem cells. Cancer Lett (2014) 349(1):67–76. doi: 10.1016/j.canlet.2014.03.035 24705306

[60] Yan Y Liu F Han L Zhao L Chen J Olopade OI . HIF-2α promotes conversion to a stem cell phenotype and induces chemoresistance in breast cancer cells by activating wnt and notch pathways. J Exp Clin Cancer Res (2018) 37(1):256. doi: 10.1186/s13046-018-0925-x 30340507 PMC6194720

